# Food, Eating, and Happy Aging: The Perceptions of Older Chinese People

**DOI:** 10.3389/fpubh.2019.00073

**Published:** 2019-04-05

**Authors:** Colette J. Browning, Zeqi Qiu, Hui Yang, Touhong Zhang, Shane A. Thomas

**Affiliations:** ^1^Research School of Population Health, The Australian National University, Canberra, ACT, Australia; ^2^International Primary Health Care Research Institute, Shenzhen, China; ^3^Department of Sociology, Peking University, Beijing, China; ^4^School of Primary Health Care, Monash University, Clayton, VIC, Australia; ^5^WHO, Beijing Office, Beijing, China

**Keywords:** food, eating, happy aging, China, older people

## Abstract

China's government and its people have for a long time focused on food security for its population as one of the most important issues in economic and social development. Many older people in China have lived in times when food security was not stable. Thus, while food has a central position in Chinese culture for all Chinese people, it is of particular pertinence to older people. In this paper we explore the meaning of food and eating in the lives of older Chinese people in China and how it contributes to healthy, thus happy aging. Focus groups and qualitative interviews were used in this study. Participants were recruited from the rural Yongfu Province of Southwest China, and the urban Fangzhuang and Haidan districts in Beijing. Forty-two participants were recruited aged 62–83 years of age. All focus groups and interviews were conducted in Mandarin and audiotaped with the participants' permission. Audio-tapes were transcribed by a Chinese speaking researcher and then were translated into English. Data were analyzed continuously and comparatively, transcripts were coded, and themes and sub-themes were identified. The initial analysis and interpretation were then presented and discussed at a workshop with all the authors. Two major themes emerged—the quantity of food and the quality of food required to have a happy old age. Participants discussed the desire to eat “until you are full” because of their experiences of famine during childhood. However, they also believed that as an older person they should eat less for their health, particularly less high fat foods. The importance of the food quality and food affordability was also discussed. Grain and meat were characterized as “good” foods and important in their diets for a happy old age. The participants, especially those from urban areas, were concerned with food safety. The high cultural importance of food for older Chinese in China was confirmed in this study. Social and economic lifespan experiences continue to impact on the food and eating attitudes and practices of older Chinese. The food related life experiences of older Chinese in China are quite different from younger Chinese and health promotion messaging needs to be informed by these unique perspectives in order to maximize its effectiveness.

## Introduction

China's government and its people have for a long time focused on food security for its population as one of the most important issues in economic and social development. Many older people in China have lived in times when food security was not stable. Thus, while food has a central position in Chinese culture for all Chinese people, it is of particular pertinence to older people (1, 2). In this paper we explore the meaning of food and eating in the lives of older Chinese people and how it contributes to healthy, thus happy aging.

Like many countries China has an aging population. In 2017, 16.0% of the people living in China were aged 60 years and over (3). By 2050 29.9% and 6.8% of the population will be aged 65 years and over and 80 years and over, respectively (4). By 2060 the number of older people in China will peak. Life Expectancy in China in 1950–1955 was 40.8 years, and by 2025–2030 it is expected to reach 78.1 years. Population aging is occurring at a much more rapid rate in China than in many other countries. For example, it took only 30 years for the proportion of the Chinese population aged 60 and over to increase from 7 to 14%, while in the U.S and France, this rate of increase took 70 and 130 years, respectively (5). Most of this rapid change in China has occurred in the history of a low GDP per capita of $US1,000, although of course China's per capita GDP has grown strongly in the last decade. The Chinese social security and health systems have many challenges in responding to this demographic change.

Countries such as Australia and the UK have responded to population aging by developing specific aged and community care services and a strong primary health care system. In addition, the call for healthy and active aging policies are championed as a way of reducing the “burden” of aging populations, and also recognize the potential of older people to age well with support, challenging aging stereotypes (6, 7). In China, such approaches have great potential as active engagement in the family and community are strong values for older people. The Chinese Government has implemented in law an official agenda for aging programs:

*All elderly people are to be provided for and enjoy proper medical care; all of them are to be given opportunities to pass on their experiences as well as to learn new things; all of them should be given the opportunity to do what they can for the society; all of them should enjoy their later years* *(**8**)*.

In order to promote healthy/active aging it is important to understand older people's perceptions of aging and their aspirations for their lives as they age (9, 10). The study reported in this paper is part of a program of research that examined older people's conceptualizations of healthy aging across a number of countries including Australia, Malaysia and China (11–13). The program asked older people to reflect on what healthy aging meant to them and to consider factors that contributed to their quality of life as they aged. In Australia healthy aging was associated with emotional bonds, physical health and well-being, maintaining independence and social interaction. The focus for older Malays was financial independence, physical health and functioning, safe living environments, spirituality and peace of mind. The broad aim of the program was to investigate: how older people understand aging and healthy aging; older people's expectations of a healthy old age; and older peoples' experiences of aging and the factors that influence healthy aging.

This current paper focuses on the role of food security in the lives of older people living in China and how this basic need contributes to their conceptualizations and experience of “happy” aging. Based on advice from our Chinese colleagues the Chinese term for happy aging was preferred to healthy aging and is used throughout rest of this paper.

The vast majority of older people living in China today have lived through a number of key events in Chinese contemporary history. These included the second Sino-Japanese War (1937–1945); the Chinese Civil War (1946–50) and the Cultural Revolution (1966–1976). Many had also lived throughout significant natural disaster events, such as the Great Chinese Famine (1956–1961). Estimates of the number of people killed by the Great Chinese Famine are in the order of 30 million (14). Poverty and food security provide the historical and social backdrop for this study.

The importance of food security as a basic physiological need was highlighted by Maslow (15):

*Anyone who attempts to make an emergency picture into a typical one and who will measure all of man's goals and desires by his[her] behavior during extreme physiological deprivation, is certainly blind to many things. It is quite true that man [people] live(s) by bread alone — when there is no bread* (1943, p.375).

According to Maslow's hierarchy of needs, until basic physiological needs are satisfied other motivating factors such as social and psychological needs cannot be addressed. Similarly, food, as a main parameter of life, was identified by the Chinese philosopher Sima Qian two thousand years ago. Based on his perspective, food is the most important need of ordinary people.

*To the ruler people are all-important; while to the people foodstuff is all-important Sima Qian, the Records of Historian, Chinese historian in Han Dynasty, 145-87 BC* *(**16**).*

In oriental philosophy, food and eating do not merely satisfy a physiological need. Food is a vehicle for family relationships, emotional well-being, and social harmony.

A number of research questions were addressed in this study:

1. How do older Chinese people in China conceptualize food and eating in their health and well-being in old age?
2. What is the importance of food and eating in the aspirations for a happy old age of older Chinese people in China?
3. What influence do early life experiences have on the role of food in happy aging for older Chinese people in China?

## Methods

Ethics approval for this study was obtained from Monash University Human Research Ethics Committee, approval number 2007000453.

Qualitative interviews and focus groups were used in this study. An inductive analysis method was used to extract themes and interpret the words of the participants in the context of the historical and political changes that have occurred in China during the participants' lives. As researchers working in the fields of aging, psychology and sociology our assumptions were that older people are willing and able to reflect on their life experiences and that their attitudes to food and eating would be impacted on their experiences across the lifespan, particularly their experiences during significant historical events such as the Great Famine. In terms of methodological approaches, our opinion is that the study research questions are most suited to qualitative inquiry. Focus group methodology allows interaction between the participants and can facilitate insights by the participants about their experiences. However, the researcher needs to ensure that each participant has opportunity to express their opinions. Where some participants preferred individual or couple interviews this was accommodated and provided triangulation of methods.

Participants were recruited for the study through local Chinese neighborhood committees and community health services personnel. These local contacts liaised with potential participants and organized a location and time for a researcher to meet with and explain the study to the participants. The participants were recruited from the rural Guilin in the Yongfu Province of Southwest China, and the Fangzhuang and Haidan districts in Beijing. Yongfu (population 268,000) is located in the northern part of Guanxi Province in the south of China. Fangzhuang is a large urban community (population 1.57 million) in the southeast of Beijing and Haidan (population 2.1 million) is located in the north west of Beijing and houses a number of universities and technology industries. These areas were selected as they provided a range of individuals from different SES backgrounds. Purposive sampling was used. Participants were women and men aged 60 years and over, from varied occupations and living circumstances (living alone, living with spouse and living with family).

A total of five focus groups were conducted. Two were conducted in Yongfu and three in Beijing. Focus groups were conducted in Mandarin by team members from PKU and Monash University. In addition to the focus groups, some face to face interviews were also conducted by the PKU research team with individuals or married couples who preferred that method of interview. Basic sociodemographic information including age, gender and occupation was collected for each participant.

The interview protocol was developed based (1) a review of the healthy/successful aging literature and (2) research team meetings with older Chinese to test the validity of the questions and feasibility of the project methodology. Questions for both the focus groups and interviews included: What words do you use to describe aging? What are the most important things to have when someone reaches old age? What does happy old age look like? How can we achieve a happy old age?

Interviews and focus groups lasted ~60 min and were audiotaped with participant permission. Audio-tapes were transcribed by a research assistant at PKU and YH translated the transcripts into English so that the English-speaking members of the team (CB and ST) could contribute to the analysis. YH used notes to explain some of the responses from participants, such as historical references or the meaning of Chinese proverbs. Backtranslation was used in a sample of the translated interviews, to ensure rigor and reliability.

Data were analyzed continuously and comparatively. The transcripts were read and re-read, phrases were coded, and themes and sub-themes were identified. Similarities and differences in the responses of the participants were identified. A workshop method was used involving all authors to present and discuss the findings and add further interpretation of the findings based on historical events that may have impacted on the participants attitudes to food and eating. The workshop allowed the identification of any differences in the interpretation of the transcripts and to revisit any key thematic areas and clarify meaning. At the conclusion of the last focus group the team concluded that data saturation was reached as no new themes were emerging.

Trustworthiness of the study was addressed using the following approaches. *Credibility* was established through the use of appropriate qualitative methods relevant to the research questions, our familiarity with the culture under investigation and our access to historical documents to interpret the impact of participants' experiences at particular times in history. Credibility was also established through the use of thick description. *Confirmability* was demonstrated by the documentation of our assumptions detailed above related to our disciplinary backgrounds and previous work with older people and our use of triangulation. In most qualitative studies, findings are relevant to specific population groups or situations so demonstrating *transferability* to other population groups or situations is not feasible. In the current study the participant's background, gender, age, occupation and living circumstances are provided and based on this information it is highly likely that the findings are transferable to others with similar characteristics.

The research findings are illustrated below using quotes from the participants.

Table 1 provides information about the age, gender, occupation and place of residence of the participants. The participants in this study came from a range of socioeconomic backgrounds and living circumstances. The participants included people living with their spouses, people living alone and people living with their children. Some had responsibility for the care of their parents, spouse or their grandchildren and others did not have any children. Formal schooling varied from as little as 3 years to university level education.

**Table 1:** Participant Characteristics.

**Participant**	**Age**	**Gender**	**Geographic location**	**Occupation**
1	60–64	M	Beijing	Retired Farm worker
2	80–84	M	Beijing	Retired Factory Worker
3	70–74	M	Beijing	Unknown
4	60–64	M	Beijing	Family massage since retirement
5	60–64	M	Beijing	Retired general manager of a large store
6	70–74	M	Beijing	Retired accountant
7	60–64	M	Beijing	Retired French interpreter
8	85–89	M	Beijing	Unknown
9	70–74	M	Beijing	Unknown
10	65–69	M	Beijing	Retired hotel worker
11	75–79	F	Beijing	Retired Kindergarten Manager
12	70–74	F	Beijing	Retired School Teacher
13	70–74	F	Beijing	Retired teacher
14	65–69	F	Beijing	Retired salesperson
15	70–74	F	Beijing	Retired bus ticket salesperson
16	65–69	F	Beijing	Retired worker
17	65–69	M	Beijing	Retired Communist Party Secretary
18	Unknown	F	Beijing	Unknown
19	65–69	F	Beijing	Retired vegetable farmer (worked in a greenhouse)
20	65–69	F	Beijing	Retired high school teacher
21	70–74	M	Beijing	Retired engineer
22	80–84	F	Beijing	Retired factory worker
23	70–74	F	Beijing	Unemployed
24	Unknown	M	Beijing	Worker
25	65–69	F	Beijing	Farmer
26	75–79	M	Beijing	Retired technician from Peking University
27	75–79	F	Beijing	Unknown
28	80–84	F	Beijing	Retired researcher and old revolutionary
29	65–69	F	Beijing	Retired farmer.
30	70–74	F	Yongfu	Farmer, still working
31	70–74	F	Yongfu	Farmer
32	70–74	F	Yongfu	Unemployed, makes her own living
33	60–64	F	Yongfu	Worked for the local government.
34	65–69	F	Yongfu	Retired financial worker
35	65–69	F	Yongfu	Retired nurse
36	70–74	F	Beijing	Has never worked outside of the home
37	60–64	M	Yongfu	Retired teacher
38	75–79	M	Yongfu	Retired veteran cadre
39	75–79	M	Yongfu	Retired teacher
40	65–69	M	Yongfu	Farmer
41	65–69	M	Yongfu	Unemployed
42	70–74	M	Yongfu	Retired worker

Forty-eight individuals participated in either an interview or a focus group. Six women in one focus group had to be excluded from the analysis because of the poor quality of one of the recorded interviews. This left a total of 42 participants, aged between 62 and 83 years old. Twenty women and 22 men participated in the interviews. Most of these (*n* = 30) lived in Beijing, with 12 from the rural Yongfu district.

## Results and Discussion

A number of themes and sub-themes concerning food, eating and a happy life in old age were extracted from the transcripts. This paper focuses on two major themes, the (1) *quantity of food* required to have a happy old age and the (2) *quality of the food* to have a happy old age. Other themes will be the subject of another paper. The themes and subthemes are summarized in Table 2. Before discussing these themes in detail, we provide a discussion of early life experiences of the participants whose attitudes to food and eating have been shaped by the historical periods they have lived through. Their descriptions of their experiences as children and younger adults, have shaped their cultural and societal values and their views of how food and eating impact on their lives in old age. The participants came from both rural and urban environments which also impacted on the way they understand the role of food in their lives.

**Table 2:** Themes and subthemes.

**Theme**	**Subtheme**
Quantity of food	Eat until you are full
	Eat less
	Eat what you want to eat
Quality of food	Eat good food
	Meat is a good food
	Food affordability
	Dietary supplements/health products
	Food safety

### Early Life Experiences: I Was Tired and Hungry All the Time

Many experienced extreme hardships as children:

*I worked in a factory since the age of 10. At that time, I was a child laborer. There was not enough food in my family. I went to school from the age of 6 to 9. I used to walk to the factory at night on foot. I worked 12 hours each night. An 8-hour day? No way! I only had one day of rest in a week* (Participant 2, 80–84-year-old Male, retired factory worker).

Others discussed their experiences with poverty, and the impact that this had on their lives and how these life experiences were important in shaping their current views about life and values in old age. Most notably, participants discussed how in the presence of poverty, family solidarity and harmony became a driving force in their lives:

*We lived a particularly difficult life. We were extremely poor. My parents had seven daughters and they raised us through hardships and tribulations. There were times when we were dying of hunger and we had to go and collect wild vegetables in the field. We worked so hard, but never had enough to satisfy our hunger* (Participant 16, 65–69-year-old Female, retired worker).

*My experience is based on my past-present comparison. I have experienced the Old Society and the New Society. I have experienced many dynasties. I have formed my conclusions based on experience. Seniors like me have a lot of life experience, and we should learn from seniors and listen to them very carefully* (Participant 2, 80–84-year-old Male, retired factory worker).

For all of the participants their early life experiences had profound impacts on their experience of old age and their views of a happy life as they age. The most salient and unforgettable past experiences of Chinese old people are hunger and poverty. Most of them lived very stressful lives when they were young. They worked hard for survival. Their limited earnings were spent on food, and this was often not enough. They are witnesses to the *Old Society* (before 1950s), in which older people had a miserable life, and some of them resorted to begging. They also witnessed the *Great Famine* (end of 1950s), where millions of people died from hunger. The experience taught them that they needed to satisfy their most basic need: obtaining food.

As well as early life experiences, life experiences across the entire lifespan, including SES experiences, also influenced the participants conception of eating and the role of food in their lives. In the last 25 years, in particular, the life of Chinese people has improved dramatically in terms of economic prosperity and the availability of goods and services. As survivors of the *Old Society* and the *Great Famine*, older people are now more satisfied with their current lives. When they compare their early lives with their lives today, the availability of adequate food and clothing contributes greatly to a happy life. The participants see this transition from famine to adequate food not only in terms of satisfying a physiological need but also in terms of social development for the whole society.

*My partner is an old worker from the Qinghe woollen cloth factory. At the time of the Japanese [invading China], [he was] just [a child] wearing buns screen [the clothes only for child, which cover the private parts of the body]. [He] Just worked here. Worked as child laborer following the Japanese invasion. After liberation, he cared if food [was]available. He is not easy* (Participant 25, 65–69-year-old, Female, farmer).

*I just think, the elders in old society [meaning: before the 1950s] had to beg for food. And think again, there was no fire to heat [the food]. If collecting some firewood, [they] needed to make fire to heat. The elders…I saw elders were suffering in my childhood. Look, I am more than 70 now and have a happy life…I saw many elders in old society…their life…misery* (Participant 11, 75–79-year-old, Female, retired kindergarten teacher).

*At that time [early 1960s] there was a grain coupon. How much grain you could buy was determined by how many grain coupons you had. It was too hard at that time. Now I don't eat much. But at that time, it was too hard* (Participant 28, 80–84-year-old Female, retired researcher).

*My partner [he died] was… a worker. I moved in with his family after marriage. Later I was tortured by his family, which hurt me a lot. I tell you, I suffered greatly. In the old society, I went to work …I did not have the right to speak. No right at all. I did not have a child after the liberation. I only had one thought in mind, I would say, just work hard, study hard, then the Communist Party will give you a bowl of food. I just started to work with this in mind* (Participant 11, 75–79-year-old, Female, retired kindergarten teacher).

### Rural vs. Urban Life

China has a traditional urban-rural dual social structure. Farmers, in ancient China experienced a much better life than urban residents. However, Chinese rural inhabitants, about 80% of the national population, were vulnerable victims of natural disasters and most current Chinese rural aged people experienced an era of famine. Unlike urban Chinese, farmers were rarely subsidized by the government. During the first three decades (1950–1970s) of the *New China*, urban workers were identified as the “leading class” and proletariat; while rural peasants were treated as a “coalition partner” of the revolution. The gap between urban and rural life was significant. Farmers engaged in heavy work, were poor and hungry. In the Communes of the 1960–70s, the collectivization further deprived farmers of their land and produce. In rural China, poverty contributed to poor food security and poor health (17).

At the end of 1980s, China's economic reform was launched in rural areas. Farmers enjoyed the “Household contract responsibility system” in which they would be able to increase their income through working on the leased land. Rural reform in China created a large increase in productivity, transitioning the farmer's experience from hunger to the availability of adequate food. Farmers were contented, mainly because the food problem was solved. The national social and economic development agenda of the Twenty-first century aims to transition China from “adequate food and clothing” to “a well-off life.” In other words, quality of life is the focus, rather than sufficient food and clothing (18).

*Our past experience? We were too tired from working in the agriculture commune. We were in the agriculture commune during our early years. Work was too tiring. [We] could not even manage to consider our children's eating and drinking. [We] could not even take care of our children. Anyway, they [children] could eat until they were full* (Participant 19, 65–69-year-old Female, farmer).

*When we were sent to the countryside [during the Culture Revolution], the salary was measured by work points. I worked hard and got more work points than other people. As there were many children in my family, they always lacked food. My family lived a comparatively hard life during those days* (Participant 31, 70–74-year-old Female, farmer).

*I feel my life is a happy life now. No worries about eating, no worries about drinking. You see… there are no worries about money now… If you want to eat beef, go buy it; if you want to eat fish, go buy it; you buy what you want to drink. Previously? If you wanted to eat something? Nothing. We were peasants. In previous years, pickled vegetable could be eaten for a full meal. In one year, or at least for half a year, we needed to eat chaff and swallow [pickled] vegetable. [Now] we don't worry about this* (Participant 19, 65–69-year-old Female, farmer).

*I just came for my husband. He had a heart attack [myocardial infarction]. I think it was caused by tiredness. Too tired. You see, on normal days there was no regulation [regular eating times]. He would not eat when it was the time for eating; not sleep when he should be sleeping; not drink when he should be drinking. You see, he was off duty at 12 o'clock or 1 o'clock, he had no time to prepare lunch. He also had a meeting, had something to say. He did not have time to eat* (Participant 23, 70–74-year-old Female, housewife).

### Quantity of Food

We now turn to a discussion of the two main themes *Quantity of Food* and *Quality of Food*.

This theme included two main sub-themes that appear contradictory ‘eating until you are full’ and ‘eating less.’ While many participants recommended eating until you are full (in part a reaction to deprivation in their early lives), they recognized that they needed to eat less ‘unhealthy’ foods such as fried foods and eat more healthy foods such as vegetables.

#### “Eat Until You Are Full”—I Have Had Enough to Eat

President Deng Xiaoping's policy in the 1980s was to solve the problem of adequate food and clothing and the Chinese government declared that the target had been reached by the end of Twentieth-Century (19). The participants expressed their happiness that they now had enough food to eat. Most Chinese, especially older Chinese who experienced hardship before their retirement, believe that “eating until you are full” means that you have a good life. Their current life is much better than their earlier lives when they had little to eat. Participants from developed areas (such as urban Beijing) to developing areas (such as the rural villages of Guilin in Yongfu county), from north China to south China, commonly agreed that there had been improvement in food security. The provision of adequate food satisfies one of Maslow's physiological needs within the hierarchy of needs pyramid. Adequate food is necessary before other needs higher up the pyramid (safety, belonging, esteem, self-actualization) can be satisfied. Equating a good life with “eating to you are full” recognizes the importance of this basic physiological need to our participants, especially given their experiences of famine in their early lives.

“Eating until you are full” does not necessarily provide good nutrition but most of the participants, especially those from rural China, believed “eating until you are full” is “good enough”; there is no need to worry about food quality.

*Yes, [I am] very happy. No worries about eating, no worries about clothes* (Participant 27, 75–79-year-old Female, retired worker).

*Enough to eat. Life is OK. Not thinking of the issue of what to eat, to buy chicken, or, to buy chicken essence* (Participant 22, 80–84-year-old Female, retired librarian).

Not all Chinese old people are beneficiaries of the national economic prosperity. Some of them still cannot afford to “eat until you are full.” Most Chinese farmers, for instance, are not eligible for retirement or to receive a pension (20). Where there are less government subsidies and the collapse of village collective funds, aged farmers have to seek basic support (including food) from traditional family-based caring and support. Therefore, “eat until you are full” is still a fundamental desire for older farmers. Rural women, who usually depend on their partners and/or children for financial support, are at greater risk of food insecurity.

There is significant variation in socioeconomic development between geographic regions in China. Rapid urbanization created bigger cities which quickly stretched into suburbs encroaching on the countryside. Between the 1950s and 2007 the number of cities in China increased from 69 to 670. Among the urban cities, 89 had a population of more than 1 million. Today more than half of the Chinese population live in cities while in the 1980s this figure was 20% (21). Many older people and their families lived in the transition areas (the urban-rural integration area) and lost the opportunity to work on farms. Their children had to find work in the cities and the tradition of family caring is facing a significant challenge due to the internal migration of younger workers. Pensions for older rural Chinese (or compensation for losing farm work opportunities) is lower (358 RMB per month) than for their urban counterparts (541 RMB per month) (22). Urbanization has brought new difficulties to older farmers who escaped from famine but now face the prospect of low income and less support from their families.

However, poverty is also an issue in the urban areas of China, particularly for older people or for families caring for older people. While many urban older people have a retirement pension, price increases for food and other consumables have put a strain on their budgets.

…*The two of us [husband and wife] are fed by one person's salary. Is it enough? Hey, that depends on the situation. How can I compare our life with others? Having a full meal [eat full] is all right. [If] no full meal? That is just the way it is. I have no way to change the situation. Situations cannot be compared; our life may be different than others* (Participant 23, 70–74-year-old Female, housewife).

*Now this [my] life could be called a happy kind of life. I do not have retirement payment; I am not eligible for a retirement pension … The monthly income is different [from person to person]. Some people get several thousand [a month]; while others get several hundred [a month]. I am not on a pension. Anyway, it is ok to have meals. For the elders in the past, there was no retirement and they had no money at all* (Participant 28, 80–84-year-old Female, retired researcher).

In addition, if their children have lost jobs as a consequence of the reform of state-owned enterprises, they may seek help from their older parents. These unemployed young people are called *ken lao zi*, a generation who depend on their parents.

*This group of young people do not have a stable job. They have no ability to solve their own financial problems. They have to rely on their parents and they create a significant burden for older people* (Participant 25, 66–69-year-old Female, farmer).

This quote reflects the tension in role of mutual/filial obligation within Chinese families. The expectation is that parents will support their children when young who in return will assist their parents as they age (23). Economic circumstances can shift the flow of support at a time when older people may be vulnerable and needing economic support from their children. While in general our participants felt that their socioeconomic circumstances and access to food had improved in their later years, those on low incomes or those who were supporting their children were concerned about food insecurity.

#### “Eat Less”—Us Older People Should Eat Less

The participants mentioned “eat less” frequently as one of their core beliefs. They agreed that they should: “Eat until we know when to stop.” Eating less (particularly fewer fatty foods) was seen as a good way of reducing the burden on the digestive system and to feel comfortable after a meal. In addition, they suggested having several small meals each day, rather than a few large ones.

…*Eating, [we] just do not eat [too] much… Eat what you want to eat today. Buy some, not much, enough is ok. Is that right?* (Participant 21, 70–74-year-old Male, retired engineer).

*[for aged people], according to my theory, we should eat… eat less… you may have an additional small meal …but you are not comfortable if you eat too much at one time when you are old* (Participant 23, 70–74-year-old Female, housewife).

*Old women, they are easy to feed … being old, we can't eat much* (Participant 16, 65–69-year-old Female, retired worker).

Some old people explained in more detail the meaning of “less.” For instance, eating less for health reasons such as less intake of oil, less intake of food containing cholesterol, or eating less meat. Some others mentioned “eating until you are half full.”

One retired worker in Beijing (participant 5, 60–64-year-old Male, retired manager of a large store), however, did not fully agree with “eat less.” He maintained that eating less and eating more are both a hazard to your health. He emphasized a “balanced diet.” He stated that: “many of us like to eat meat; we would like to have meat in every meal.” He concluded “we cannot accept meat free meal; while we also cannot endure greasy food.”

While talking about “eat less,” participants also suggested eating more of some foods. Vegetables and fruits were the most common foods they believed should be eaten more often. Participants also specifically promoted eating more “coarse foods,” such as corn.

*Which things are most important [for happy ageing]? Just playing well and eating well are the most important. First you pay more attention to eating and drinking for your diet. Eat less, but more often, but not more than six meals [per day]. Is that right? Eat more vegetables, eat more coarse cereals. Eat less oil. Those foods with a lot of oil should be eaten less* (Participant 17, 65–69-year-old Male, retired village carder).

*I like to eat meat. Meat should be in each meal. Otherwise your health will be poor. [Some diseases are] caused by eating [too much or too little meat]… In my fried beans [dish], I just need a meat taste, a small amount of meat. [I] have hypertension, also my age is high [nian ji da le, means become much older], I should eat less meat. Look, I listen to TV and the elders say, elders should try to eat less meat* (Participant 36, 70–74-year-old, Female, retired worker).

#### Eat What You Want to Eat

Our participants believed that eating what you want ensures a happy and satisfying old age. This belief is related to their ability to independently choose what they want to eat.

*To take care of ourselves, that is enough. Stay happy and glad for the whole day. Eat what you want to eat. That is enough… I have good health through physical exercise. Another thing is to be happy. To play what I can play, to eat what I can eat. It is simply that* (Participant 21, 70–74-year-old Male, retired engineer).

*My favorite meal is just steamed buns and rice, and some stir fried dishes. Then… it does not matter what else* (Participant 21, 70–74-year-old Male, retired engineer).

*I know a cancer [patient]… He is back at home and eats what he wants to eat, then exercises the Happy Kungfu … He is nearly recovered… Now his health is good* (Participant 21, 70–74-yearold Male, retired engineer).

*Eat what you want to eat…it is important for your condition* (Participant 17, 65–69-year-old Male, retired architect).

In summary, our participants agreed that today, because food is more readily available than when they were young, they are able to “eat until they are full.” However, they believe that as an older person they need to have smaller meals and avoid foods that are “unhealthy” such as fried foods and eat more “healthy” foods such as vegetables.

Our findings are consistent with similar studies of habits and beliefs of older Chinese. In a qualitative study of Chinese-American older women, participants noted the importance of a balanced diet for health, eating regular simple meals, and maintaining a stable weight. The consensus from the participants was that a healthy diet should include vegetables and fruit, “eating less meat,” not eating too little or too much, and avoiding high fat foods (24). A study in rural China highlighted grandparent's concerns about not having enough food for their grandchildren and the avoidance of hunger based on their experiences in the Great famine (25). A Hong Kong study highlighted that older people were concerned about overeating fatty or fired foods and understood the role of healthy eating, including eating fruit and vegetables, in maintaining good health (26).

Our findings are also consistent with Western studies of older people and their eating beliefs and habits. For example, in an Australian study, older adults valued eating well for health and acknowledged the influence of childhood patterns of eating on current patterns (27).

Our participants also discussed food quality.

### Quality of Food

#### Eat Well, Eat Good Food, Eat Simple Food

Participants often maintained that they should “eat well,” “eat better,” or “eat good food.” This is in part an indication of their desire to eat good quality food and food that is good for their health.

*We are now retired and stay at home. We have a willingness for a relaxed life, a little bit of relaxation, not too busy like in the past. We would like to slow down the rhythm of life a little bit, slow down a little…In financial terms, we also want to be a little bit richer. Except for subsidizing, the children and the grandson, we also would like to eat better, and dress better* (Participant 12, 70–74-year-old Female, retired high school teacher).

However, there is variation in what is meant by “good food” in Chinese culture and these different meanings are described below (28).

*Expensive food* may be judged as good food. For instance, before the 1980s, the price of corn was much lower than that of wheat, and wheat was less available than corn. Therefore, wheat was recognized as a “good food.” In recent years, the price of corn has increased and exceeded the price of wheat. *High fiber foods* derived from wheat and corn are considered “good foods.” *Scarce foods* are also considered good foods. During the Great famine meat was virtually unavailable to most of the population. Therefore, meat is seen as a “good food.” *Restaurant food* is considered good due to the skill of the chef, the physical environment and the community atmosphere. *Enjoyable or satisfying food*, or *food that is easy to digest* is considered good food. *Homemade food* enables the person to satisfy their personal preferences and often is eaten in a family environment and is therefore considered good food. Food that is consistent with what was eaten when they were younger is considered good food and is often related to local legends such as “birds are more tasty than beasts,” or with eating *simple food* associated with their childhood.

Among these descriptions about what is “good food,” the most popular one for the participants in this study is *cu cha dan fan*, literally translated as “ordinary tea and a simple meal,” or in Western terms, “a simple diet.” Their judgement on good food quality is closely related with their previous life experiences. They are easily satisfied because the food quality was so poor in their childhood. Simple food is good because it evokes memories of their childhood, their struggle, and joys and sorrows of their life.

*I also pay attention to my diet, because like us, we all had a hard life previously, especially those who went through the hard times [meaning the years of severe natural disasters in the 1960's]. Many people died from starvation; grain was very tight. There were many siblings in my family, and life was also difficult. Therefore, we usually had a simple diet, the most normal diet. If you really let us eat chicken, duck, meat, fish, I feel, we will be tired of this after several meals, and will not be used to it. I just like to eat the green vegetables from our home town… that is a simple meal* (Participant 20, 66–69-year-old Male, retired high school teacher).

*When our prime minister visited four seniors in Shanghai, each of them told him the secret to longevity. One of them said ‘green tea and simple food’, which symbolizes a simple life. Simple as his life is, he was never haunted by hunger. He kept a balanced diet, ate very simple food, but more vegetables. So that explains it. And we do not seem to have much to care about, do we?* (Participant 37, 60–64-year-old Male, retired teacher).

#### Meat as Good Food

In China, vegetarians are minorities and most of those refuse to eat meat for religious reasons. The *Nirvana Sutra* of Buddhism suggests “to eat meat will break off root of mercy.” However, it seems not all believers of Buddhism do not eat meat. In the three main streams of Buddhism, the monks of Chinese Buddhism claim to be meat free; while Theravada and Tibetan Buddhists can eat “clean meat.” The traditional Chinese religion, Taoism, also recommends a no meat diet, however, only Taoists of Quanzhen Taoism refuse to eat any meat (29).

In contemporary mainland China, the majority of people claim no religious affiliation. Among the Chinese general population, foods containing meat and/or oil have been traditionally described as “good food.” Children and teenagers are encouraged to eat more meat in order to promote physical development.

There are many Chinese idioms and legends about meat. For instance, *jiu rou peng you* (good friends are those who can share meat and wine), *rou shi zhe bi* (people who eat meat are shallow, where the people are from the elite). “Plenty of fish and of plenty meat” (*da yu da rou*) is a metaphor for being rich and attaining high social status; while “behind the vermilion gates meat and wine go to waste while out on the road lie the bones of those frozen to death” (*zhu men jiu rou chou lu you dong si gu*) is a ballad from Old China, a snapshot of the difference in food security between rich and poor (30).

As discussed previously, most of our participants experienced the Great Famine at the end of 1950s. At that time, the participants were teenagers or young adults. They lived in either urban or rural areas and survived extreme poverty. Eating meat was an extreme or unusual event. In addition, most families used animal oil/fat for cooking because vegetable cooking oil was unavailable at that time. In rural China, there was virtually no meat consumed. Most people had food with meat or fried food only at their family reunion dinner, the supper celebrated on Chinese New Year's Eve.

Those traditions and experiences significantly impact on the belief that “meat is good food” in Chinese culture and as such meets a physiological need.

*I was in the countryside for decades and married a teacher at that time…lived in the countryside when I was nineteen years old. … At that time, meat was bought with meat vouchers. [In the early years of liberation, many daily necessities such as rice, eggs, meat, clothes were purchased with vouchers]. In that period, a family had many children and only a limited number of vouchers. So, they dissolved fat meat into liquid lard and used it for cooking. As soon as the children came back, they wanted to eat the meat from which fat had been extracted. But it was not enough for them. I think those were tough days and I shed tears many times* (Participant 31, 70–74-year-old, Female, farmer).

However, the participants suggested that eating this “good food” should be moderated: “Meat is delicious but should be eaten less.” They believe that to eat too much meat will be bad for their health. Other studies of dietary practices of Chinese older adults have noted the competing values concerning the consumption of meat: there is a preference to eat meat but participants understand that too much meat, particularly fatty meats can cause health problems (24, 25).

*In the past, we wanted to eat meat but there was no meat to eat. Now we can eat meat at any time, but we should not eat too much* (Participant 32, 70–74-year-old Female, unemployed).

*I do not like eating chicken, duck and fish, and I would not like to eat those things. Considering the fact that only my wife and I live at home and our children do not live with us, we only need to prepare meals for ourselves, two aged persons. That is enough for us. We do not eat meat every day. Now things are different than in 1956, a hard year, when there was no meat or fish to eat. Currently, if you want to eat meat, there is enough meat on the market. However, some people do not like or do not want to eat meat* (Participant 34, 65–69-year-old Male, retired financial worker).

*Too much fat and meat in the diet will affect health, but it is ok to have some comparatively good food. This is what I really think of… diet. A light diet without much meat and… fish is good for your health* (Participant 33, 60–64-year-old Female, local government worker).

*I have a part time job, looking after children, cooking meals … ‘What do you like to eat?’ my children always say to me, ‘You do not have regular meals’… look, sometimes I eat less, sometimes I eat more… I normally have rice… Only during the winter do I like to eat Chinese cabbage. I like to eat fried capsicum. I like to eat garlic bolt. My teeth are not good, I cannot chew. I do not like eggplant… I do not eat meat on hot days, I just eat less meat…Young people cannot be without meat to eat. If you [young people] do not eat meat, you cannot keep up. If you do not eat meat when young, your strength cannot keep up. But you cannot just eat meat* (Participant 14, 65–69 Female, retired salesperson).

#### Food Affordability

Before the 1980s in China, food availability impacted on what older people could eat. Since the 1980s the Chinese food market has developed in terms of quantity and quality with accompanying increases in the price of food. Affordability of food is a key determinant of older people's diet. Food price increases are the main contributor to CPI increases in China According to the Chinese National Health Service Survey (2003), food expenditure comprised 44.4% of total family expenditure in urban families, while the proportion was 37.0% in rural families. Based on the survey, family expenditure on food as a proportion of total family expenditure decreased by 10% between 1998 and 2003 (31). Family expenditure on education and medical care increased dramatically over that period. More recently, the proportion of household expenditure on food has further decreased to 36.2 percent (32).

The Chinese government is acutely aware of the impact of price rises on people's lives, especially those who are retired and on low incomes. The retirement pension and low-income allowances are adjusted frequently, to support a good quality life for retired people but the participants were very concerned about food price rises (22).

In this study, participants often commented that their pension or allowance was just for food or was used as insurance for unexpected events. Significant uncertainty or unexpected expenses in the future (for example medical care) drove older people to save money from their pension or allowance. If the price of food increased the participants would spend less on food. Participants expressed their worries concerning increasing food prices. The following participants reflect on price rises in the past and present.

*Yes, I know everything. I can tell you some past stories. Three dynasties… Japanese is a dynasty, Kuomintang is a dynasty, the Communist Party is a dynasty. Let us talk about Japan. I have full experience in the Japanese dynasty. I had dealings with the Japanese, worked for the Japanese, and was bullied by the Japanese. Let us talk about Kuomintang… the most unforgettable was that we ate ‘mixed flour’. In the Kuomintang period, rising prices happened so fast! Skyrocketing! The Kuomintang did something… gave you more salary… adjusted the salary many times a month. You can buy a bag of rice today; however, you might buy a half bag of rice tomorrow. The price increase was too fast. So, up to now, I am scared when prices rise. Although our country is stable now, prices of food and oil are increasing, seriously. Am I right? Of course, the current price increase is not as serious as that of the Kuomintang. But that is reality… that is reality. Do you agree with me? In the Kuomintang, the government printed big bills… a bill of a million dollars, a bill of 10 million dollars… The price rise was almost the same in the early years of the New China. The Communist government launched many programs to provide adequate clothing and food to people. But, saying it politely, the problem was not solved. In 1960 (the Great Leap Forward) bad climate conditions caused the Great Famine, it was the most difficult time for us. We had to find waste vegetables to eat. We had to eat steamed corn bread] every day. That was the most difficult time. I have experienced that. Those past dynasties, I remember all the details… about the ordinary situation, the community in general. I am afraid the situation will happen again* (Participant 2, 80–84-year-old Male, retired factory worker).

*We just think we have not got what we particularly want. Now the vegetables and the fruits are all too expensive* (Participant 12, 70–74- year-old Female, retired high school teacher).

#### Dietary Supplement Products

During the last two decades, the dietary supplement market (vitamins, herbs, protein supplements) in China has grown rapidly (33) and further growth is expected. The China Healthcare Association Health Food Market Working Committee recently valued the health food market in China at US$30 billion. It forecasted that the market will grow 10 per cent year-on-year between 2015 and 2025 (34).

Many of these so-called health products focus on the aging population. Marketing of these products is directed to “buying health” but the market is poorly regulated. In the mid 1990s the dietary supplement market was buoyant in China but by the end of the decade questions concerning the quality of the products, and exaggerated and false claims regarding their efficacy, impacted the market. The arrival of “trusted” Western brands has seen an increase in the market (34, 35).

Participants from Beijing expressed their distrust of those health products. The believed that the products provided nothing to improve their health. More than half of the participants who used health products did so because others sent the products to them as gifts. Around only one third of those using health products brought the products themselves.

Chinese aged people do need accurate and relevant information about food and food supplements for maintaining and promoting their health. However, many of our participants distrust the claims of those marketing dietary supplements.

*Of course, health is the most important, definitely! Old people have to protect their health. It is meaningless to show how much money you have. If you are in bad health, all of the money is useless. There is a lot nonsense on the media [rubbish or gibberish messages]. There are many “health care products" on the market. Can we maintain our health through eating those “health care products"? I think that is nonsense. I think everyone knows his [or her] way of maintaining their health. They know how to protect their health* (Participant 2, 80–84-year-old, Male, retired factory worker).

*Ok, let me say… for instance, in life (note: life style), I think ‘Cha Bu Duo’ [literally, no significant difference, no more than enough, better but not the best] is appropriate. Over nutrition has no benefit. Am I right? I think Cu Cha Dan Fan (literally, cheaper tea and simpler food, plain food) is always good. If needed, you can add some meat or milk (to your meal). I do not trust the ‘health care products’. All the products are useless* (Participant 2, 80–84-year-old Male, retired factory worker).

#### Food Safety

Food supply has been dramatically improved since the 1990s in China. Chinese people can buy any food and vegetables across the year. Before the 1990s, both rural and urban families stored and ate Chinese cabbages only during the 3 months of winter.

Food safety was considered a concern by the participants, particularly those from Beijing, rather than food availability. Many participants worried about the safety of food and stated that the safety issue could be a barrier to accessing essential foods for their health. They worried about foods containing high levels of fertilizers and pesticides, although they were aware that the government had stated that those hazards were lower than the required minimum standards (36).

In the recent decades, many contaminated foods were reported in China's media. Food producers and suppliers added illegal chemicals for improving or maintaining food appearance and taste. The contaminated foods usually had better presentation and a higher price. Consumers, especially older people, have insufficient information about food safety The participants wanted the government to strengthen food inspection and surveillance. With their increased worries about food safety, the participants tried to seek out safe food or avoided food reported in the media as contaminated (37).

*How to say, the Nation [government] says it checks [food quality] everyday, it doesn't work. My son bought a basket of apples. After they were opened, [the apples were] red outside, all are sticky… At the end when he was not in front of me, I secretly threw them into the rubbish. I was afraid that when he was back [he would] scold me, say that I am wasteful. Indeed, the apples can't be eaten at all* (Participant 12, 70–74-year-old Female, retired high school teacher).

*Vegetables and fruits can give us diarrhea after eating* (Participant 12, 70–74-year-old Female, retired high school teacher).

*The food… currently… I mean… none of the food is assured and safe. We have to select simple… the simplest food. Cu Cha Dan Fan [literally, raw tea and pure rice. It is similar to the concept of ‘bread and water’] is enough for us, and we feel it is safer. Do you know the ‘Hainan Banana Event’? That banana [shipped from Hainan Province of China]! Can you eat that banana? The banana was soaked in some poisonous chemical liquid…to maintain freshness! What a taste… a puckering taste! I know officials can obtain safe foods, because they have special suppliers. Chemical fertilizers, pesticides, genetically modified… do you think these foods are good enough? At least, I think the foods are unsafe* (Participant 1, 60–64-year-old Male, retired university worker).

*I do exercise every day. In the morning, I pick or plant some vegetables of my own. It is safe to eat my own vegetables, which contain no residuals of pesticides. In the evening, I dance or walk in the square after supper* (Participant 35, 65–69-year-old, Female, retired nurse).

## Study Strengths and Limitations

This study uses qualitative methodology and as such cannot provide information about the frequency of the views expressed by the participants nor how frequent these views would be in similar populations. However, the study sample is large and includes a range of participants from different SES backgrounds. We sampled from rural and urban areas but due to the geographical diversity of China we may have missed groups living in more remote areas and this potentially impacts on the transferability of our findings. In addition, we did not sample from minority ethnic groups in China so our findings may not be relevant to these groups. The views of the participants are rich in detail and we were able to extract in-depth information about their food habits, beliefs and preferences and link these to their early life experiences and current circumstances. Translation of transcripts into English for English readership can be problematic as meaning can be lost. Three of our team members are native Chinese speakers and through back-translation and our analysis workshops we were able to use their skills in interpreting the meaning of the transcript text.

## Conclusion

In this study we explored the meaning of food and eating in achieving happy aging for older Chinese people. This qualitative research project involving older Chinese people discussing the issues they identified in their own terms. The results clearly demonstrate that food and eating rituals are perceived as very important components of the enjoyment of a happy old age. The study fits within the tradition of using qualitative methods to interpret healthy eating. As noted by Bisogni et al. (38) p. 282:

‘*The rich descriptions and concepts generated by qualitative research can help practitioners and researchers think beyond their own experiences and be open to audience members’ perspectives as they seek to promote healthy ways of eating.’*

The collective and personal histories of older people in China described by the participants show they have experienced significant changes across their lifespans in the quantity and quality of food. These changes have been closely linked to their well-being and economic prosperity. In 1960 life expectancy at birth in China was 43.7 years and in 2018 it is 76.4 years. Some of the participants had experienced extreme hunger in their early lives but now were able to eat “until they were full.”

However, the participants also recognized that overeating and inappropriate unhealthy eating could be a problem for their health. The quality of food was important to them and many discussed the merits of eating simply and the role of “good” foods such grains and previously scarce foods such as meat. Despite the increased general availability of food, affordability was a key issue for many of the participants, as was food safety. In this article the different meanings of “good food” are compared and discussed. These provide important insights into the varying conceptualizations of food.

The study has demonstrated that social and economic lifespan experiences continue to impact on the food and eating attitudes and practices of older Chinese. It is important for health professionals working with older Chinese to understand these impacts and provide health education and promotion approaches to maximize healthy food choices that align with the aspirations of older people to achieve happy aging. The food related life experiences of older Chinese are quite different from younger Chinese and health promotion messaging needs to be informed by these unique perspectives in order to maximize its effectiveness.

## Ethics Statement

This study was carried out in accordance with the recommendations of Monash University Human Research Ethics Committee with written informed consent from all subjects. All subjects gave written informed consent in accordance with the Declaration of Helsinki. The protocol was approved by the Monash University Human Research Ethics Committee.

## Author Contributions

CB, ST, ZQ, HY, and TZ led the conception of the study. All the authors obtained research and operational funding. CB and HY drafted the paper and all authors contributed to critical revision. HY led the data analysis and all authors contributed to the interpretation of the data. All the authors had full access to the study data and take responsibility for the integrity of the data and the accuracy of the data analysis. All the authors have approved the final version of the manuscript.

### Conflict of Interest Statement

CB, ST, ZQ, and HY are Editors of the research topic: Chronic Illness and Aging in China; they had no involvement in the review of this manuscript. The remaining author declares that the research was conducted in the absence of any commercial or financial relationships that could be construed as a potential conflict of interest.
